# Molecular characterization and functional expression of flavonol 6-hydroxylase

**DOI:** 10.1186/1471-2229-4-20

**Published:** 2004-12-13

**Authors:** Dominique Anzellotti, Ragaï K Ibrahim

**Affiliations:** 1 Molecular Membrane Dynamics Laboratory, Department of Anatomy and Cell Biology, McGill University, Montreal, H3A 2B2, Canada; 2 Plant Biochemistry Laboratory and Center for Structural and Functional Genomics, Department of Biology, Concordia University, Montreal, H4B 1R6, Canada

## Abstract

**Background:**

Flavonoids, one of the major groups of secondary metabolites, play important roles in the physiology, ecology and defence of plants. Their wide range of activities is the result of their structural diversity that encompasses a variety of functional group substitutions including hydroxylations. The aromatic hydroxylation at position 6 of flavonols is of particular interest, since it is catalyzed by a 2-oxoglutarate-dependent dioxygenase (ODD), rather than a cytochrome P450-dependent monooxygenase. ODDs catalyze a variety of enzymatic reactions implicated in secondary metabolite biosynthesis.

**Results:**

A cDNA fragment encoding an ODD involved in the 6-hydroxylation of partially methylated flavonols, flavonol 6-hydroxylase (F6H), was isolated and characterized from *Chrysosplenium americanum *using internal peptide sequence information obtained from the native plant protein. This novel clone was functionally expressed in both prokaryotic and eukaryotic expression systems and exhibited ODD activity. The cofactor and cosubstrate requirements of the recombinant proteins are typical for ODDs, and the recombinant enzymes utilize 3,7,4'-trimethylquercetin as the preferred substrate. The genomic region encoding this enzyme possesses two introns at conserved locations for this class of enzymes and is present as a single copy in the *C. americanum *genome.

**Conclusions:**

Recombinant F6H has been functionally expressed and characterized at the molecular level. The results demonstrate that its cofactor dependence, physicochemical characteristics and substrate preference compare well with the native enzyme. The N-terminal region of this protein is believed to play a significant role in catalysis and may explain the difference in the position specificity of the 6-hydroxylation reaction.

## Background

**Figure 1:**
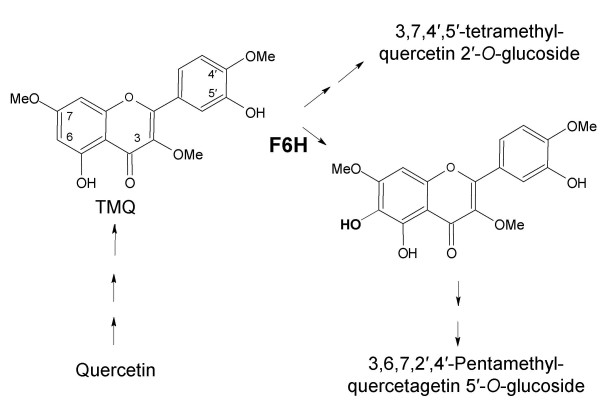
Proposed pathway for polymethylated flavonol biosynthesis in *C. americanum*

Flavonoid compounds constitute one of the major groups of secondary metabolites and play important roles in plant development, reproduction and defence. This diverse spectrum of activities results from their structural diversity and a variety of functional group substitutions [1]. *Chrysosplenium americanum *(Saxifragaceae), a semi-aquatic weed, accumulates a variety of tetra- and penta methylated flavonol glucosides substituted at various positions of the flavonol ring [2]. Their biosynthesis from the parent aglycone, quercetin, is catalyzed by a number of stepwise, substrate-specific, position-oriented *O*-methyltransferases and two distinct *O*-glucosyltransferases in a highly ordained sequence (Figure 1) [2]. During the course of their biosynthesis, the partially methylated intermediate, 3,7,4'-trimethylquercetin (TMQ) is hydroxylated at position 6 to 3,7,4'-trimethylquercetagetin (TMQg) by a 2-oxoglutarate-dependent dioxygenase (ODD), flavonol 6-hydroxylase [3].

Plant ODDs (EC 1.14.11.-) constitute a class of non-heme, iron-containing cytosolic enzymes that utilize an oxoacid as a cosubstrate and a reducing agent for the reactive iron moiety, typically ascorbate. This widespread class of enzymes has been implicated in a variety of plant metabolic pathways, including the biosynthesis of some amino acids, hormones, signalling molecules and a variety of secondary metabolites [4]. Hydroxylation at position 6 of partially methylated flavonols is of particular interest, since it is catalyzed by a 2-oxoglutarate-dependent dioxygenase rather than a cytochrome P450-dependent monooxygenase.

Using the peptide sequence information obtained from the purified plant protein [3], degenerate primers were designed for the screening a *C. americanum *cDNA library and the isolation of a F6H clone. The identity of this clone was confirmed after identifying internal peptide sequences in the translated amino acid sequence of the isolated cDNA clone, as well as by functional expression of F6H in both prokaryotic and eukaryotic systems. The molecular characterization of this gene, with respect to its genomic organization and phylogenetic relationship to other ODDs involved in plant secondary metabolism, contributes to the growing pool of information on this class of enzymes.

## Results and discussion

### Isolation and cloning of F6H

Several peptides were obtained from tryptic digestion of the native protein, one in particular, Micro1 – DNGWILLHIGDSNGHR, exhibited significant similarity to other known flavonoid ODDs such as the flavanone 3-hydroxylase (F3H) [5], flavonol synthase (FLS) [6] and anthocyanidin synthase (ANS) [7]. Two other peptide sequences, Micro2 – KIVEACEDWG and Micro3 – TLMAGLACKLLGVL, exhibited limited homology to this class of enzymes. Of the different approaches used, two methods allowed the isolation of putatively positive cDNA fragments from a *C. americanum *bacteriophage expression library [8]. The PCR-based strategy utilized several primer combinations and resulted in the isolation of a fragment termed, F6H·1, that exhibited a significant degree of homology to flavonoid ODD genes. In addition, a portion of the deduced amino acid sequence of F6H·1 matched another fragment obtained from microsequencing. Subsequently, the PCR-based screening approach resulted in the amplification of a 1,245 bp-long cDNA fragment, termed *cF6H*, that contained the 3'-end of this putative cDNA clone, including an in-frame stop codon (TAA), a putative polyadenylation signal (ATATAA) and a short polyA tail (Accession # AY605048), although it lacked the translation start site. The same cDNA library was also screened with an oligonucleotide probe derived from the F6H·1 sequence. Putatively positive clones were isolated and characterized, but none yielded a full-length F6H cDNA clone, although several clones were isolated with homology to related sequences, particularly *F3H *and aminocyclopropanecarboxylate oxidase *(ACCO)*. Further attempts to isolate a complete F6H ORF were unsuccessful, including the use of inverse PCR and the GenomeWalker technique (ClonTech).

**Figure 2:**
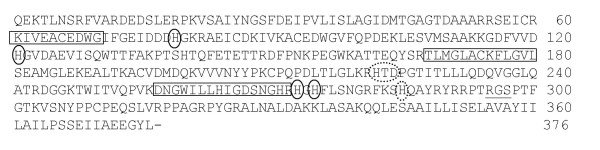
Translated *cF6H *sequence. Illustrates the conserved residues involved in iron binding (dashed circles), cosubstrate binding (underlined), other strictly conserved residues (encircled) and location of internal peptide sequences (boxed).

The truncated DNA fragment, *cF6H*, translates into a 376 amino acid-long sequence with a predicted molecular mass of 40.9 kDa and a *pI *of 5.1. The fact that the native protein exhibits a molecular mass of 43 to 45 kDa following gel filtration or SDS-PAGE analysis respectively [3], suggests that the cDNA fragment lacks ~10 to 15 N-terminal amino acid residues. However, the conserved regions and catalytically important residues located in the C-terminal of ODDs [9] are present in the translated *cF6H *sequence. The amino acid residues involved in iron (His222, His285, Asp224) and α-KG-binding (Arg295, Ser297), as well as other conserved residues are present within the *cF6H *sequence (Figure 2). The near-full length *cF6H*, although truncated at the N-terminal, was confirmed to be an authentic fragment of the gene encoding the F6H, since the internal peptide sequences obtained from the native protein were present in the deduced amino acid sequence of the cDNA clone (Figure 2). Sequence analyses of ODD genes including *cF6H *demonstrate that the conserved regions and catalytically important amino acids are located primarily within the C-terminal half of these proteins, whereas the less conserved N-terminal region may be involved in substrate binding or maintaining the protein's tertiary conformation. Given the inherent variability of the N-terminal, that in certain cases it does not contribute significantly to ODD enzyme activity as was the case with the desacetoxyvindoline 4-hydroxylase of *Catharanthus roseus *[10], and that all possibilities for the isolation of the remaining fragment were exhausted, the *cF6H *fragment was cloned into prokaryotic and eukaryotic expression vectors to assess its enzyme activity.

### Expression of recombinant F6H

**Figure 3:**
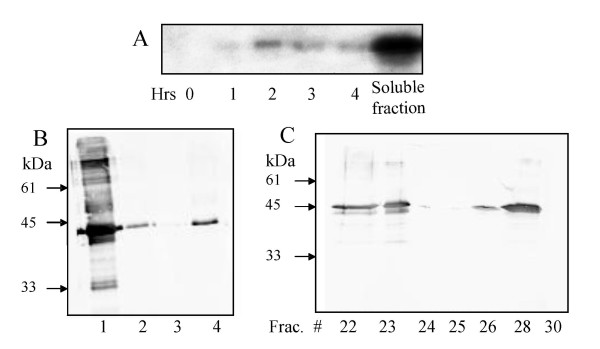
rF6Hb expression as detected by immunoblotting using anti-His antibody (A) Time course induction of rF6Hb expression. Soluble fraction represents total cell lysate. (B) Purification of rF6Hb on Ni-NTA; Lanes: 1, flow-through (10 μg); 2, wash (5 μg); 3, Bound 1 (1 mL eluate, 2 μg); 4, Bound 2 (2 mL eluate, 2 μg). (C) Purification of rF6Hb eluted from Superose 12 (10 μg/fraction).

The induction and solubility of the bacterial expressed F6H (rF6Hb) fusion protein were assessed over a 4-h period and recombinant F6H enzyme activity was assayed. The level of expression of the fusion protein was monitored by Western bolt analysis of cell lysates using anti-(His)_6 _antibodies. The antibody reacted with a 45 kDa protein band in cell lysates carrying the pTrc-His-cF6H construct after induction (Figure 3). This corresponds to the correct mass of the recombinant protein since the (His)_6_-tag and extraneous amino acids resulting from the cloning process account for an additional ~3.5 kDa in the fusion construct. The Ni-NTA-purified recombinant protein (Figure 3) exhibited a F6H activity of 0.103 pkat/mg following size exclusion chromatography (Table 1). The level of enzyme activity did not vary significantly regardless of the time or temperature of induction.

In order to test for any inhibitory effect resulting from the (His)_6_-tag, the fusion protein was cleaved by incubation with enterokinase (EK). The enzyme activity of the cleaved protein was significantly reduced compared to the Ni-NTA-purified rF6Hb (Table 1). The loss of activity was attributed to destabilization of the protein resulting from dialysis to remove inhibitors of the EK reaction, followed by incubation at room temperature with EK. Control experiments indicated that the (His)_6_-tag did not stimulate overall recombinant enzyme activity, since the intact rF6Hb treated in parallel with the preparation undergoing cleavage exhibited a similar reduction in enzyme activity.

**Figure 4:**
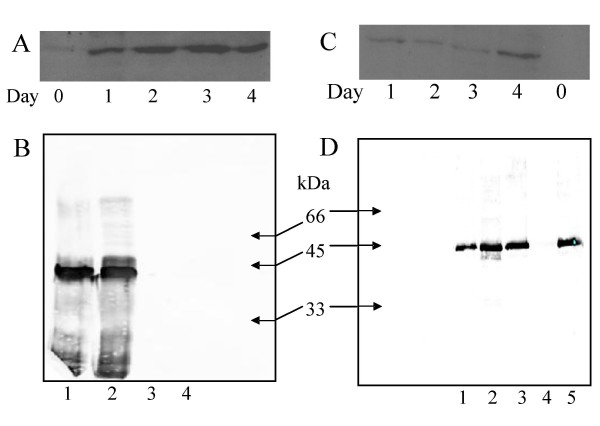
Expression of rF6Hy/rF6Hys as detected by immunoblotting using anti-His antibody (A) Time course induction of rF6Hy. (B) Purification of rF6Hy on Ni-NTA. Lanes: 1, Bound 2 (2 mL eluate; 5 μg); 2, Bound 1 (1 ml eluate; 5 μg); 3, wash (10 μg); 4, flow-through (10 μg). (C) Time course induction of rF6Hys. (D) Purification of rF6Hys on Ni-NTA. Lanes: 1, Bound 3 (3 mL eluate; 2 μg); 2, Bound 2 (2 mL eluate; 2 μg); 3, Bound 1 (1 mL eluate; 5 μg); 4, wash (10 μg); 5, flow-through (10 μg).

The translated *cF6H *sequence possesses two potential glycosylation sites at Ser139 and Ser305 that may contribute to catalytic activity, perhaps by stabilizing a favorable tertiary conformation or through the modulation of interactions with other proteins. The effects of such post-translational modifications and protein folding were taken into account by expression of the recombinant protein in the eukaryotic expression system *Pichia pastoris*. This system is relatively easy to manipulate and exhibits many of the advantages of eukaryotic expression, including protein processing, folding and modification. Recombinant protein expression was both intracellular (rF6Hy) and secreted (rF6Hys). The chosen culture media (BMGY/BMMY) contained protein stabilizing factors, such as peptone and yeast extract, shown to reduce proteolysis, particularly of secreted proteins [11]. Protein expression, monitored by Western blot analysis using anti-(His)_6 _antibodies, was maximal at 3 to 4 days post-induction in both intracellular and secreted systems (Figure 4), and the protein eluted at a volume corresponding to ~40 kDa from a Superose 12 column. The Ni-affinity-purified preparations exhibited a specific activity of 0.21 and 0.11 pkat/mg for the intracellular (rF6Hy) and the secreted (rF6Hys) constructs, respectively. Their specific activities could not be further improved by size exclusion chromatography instead of or in addition to, Ni-NTA purification (Table 1).

In both prokaryote and eukaryote expression systems rF6H was soluble, exhibited similar physicochemical properties to the native protein and was no more susceptible to proteolytic degradation. Interestingly, as with the native plant protein [3], the recombinant F6H was functional as a monomer, but was also shown by gel filtration to associate *in vitro *(Figure 3C). In the case of the native F6H, this may result from the disruption during isolation of any weak interactions with other proteins, particularly those involved in polymethylated flavonol biosynthesis. A hydrophobic prediction plot of the translated *cF6H *(data not shown) indicates the presence of a nonpolar region at the very end of the protein sequence, which may participate in dimerization or aggregation processes.

The substrate specificity of recombinant F6H was tested using the Ni-NTA-purified enzyme with different flavonol substrates (Table 2). The results indicate that, although the overall specific activity of both recombinant proteins is reduced as compared to the native protein, 3,7,4'-TMQ is the preferred substrate for both rF6Hb and rF6Hy, which accepted neither quercetin nor 3-methylquercetin to any significant extent. On the other hand, 3,7-dimethylquercetin was accepted at 56% and 63% of the control activity by rF6Hb and rF6Hy, respectively.

Given the fact that neither of the recombinant proteins utilized naringenin as a substrate, it seems unlikely that F6H catalyzes a side reaction of F3H or a bifunctional activity as F3H-FLS, as has been demonstrated with the *Citrus unshiu *bifunctional dioxygenase [12]. Therefore, this enzyme may putatively be classified as a narrow-specificity ODD [12]. Taken together, these results suggest that *Chrysosplenium *F6H exhibits a specificity for partially methylated flavonoids involved in polymethylated flavonol biosynthesis characteristic of the species.

The cofactor requirements for the bacterially expressed recombinant protein were similar to those of the native plant protein [3]. Ferrous ions had the greatest effect on enzyme activity and enzyme reactivation in affinity-purified samples, as demonstrated by abolition of enzyme activity upon incubation with 5 mM EDTA. A preliminary assessment of the apparent *K*_*m *_values of rF6Hb for the flavonol substrate (63 μM) suggests that the binding affinity has been reduced 17-fold compared to the native protein (0.27 μM) [3]. In contrast, that for α-KG was only slightly affected (78 μM instead of 60 μM), indicating that the binding site for the cosubstrate has not been substantially altered. Nevertheless, a detailed kinetic analysis of the recombinant protein was not conducted given the truncation of the N-terminal and reduced enzyme activity.

The hydroxylation of an aromatic carbon may be the result of substrate positioning in relation to the oxidizing moiety within the active site. Mehn and colleagues [13] in attempting to elucidate the role of the cosubstrate in oxygen activation by ODDs through the use of synthetic iron complexes have also demonstrated the hydroxylation of phenolic substrates. In addition, the nature of the substituents on the phenolic moiety, dramatically affects the reaction rate. These results suggest that the structure of the substrate and its positioning within the active site play a crucial role in aromatic hydroxylations by ODDs as opposed to more fundamental differences in reaction mechanisms. This may explain the importance of the N-terminal to F6H enzyme activity, particularly if it is involved in substrate binding either by directly contributing to the flavonol binding site, or by maintaining the appropriate conformation for substrate recognition. This hypothesis is reinforced by the fact that rF6H exhibited a reduced specific activity in comparison to the native F6H, regardless of the expression system, degree of purification or the location of the polyhistidine tag.

### 3.2 Molecular characterization

**Figure 5:**
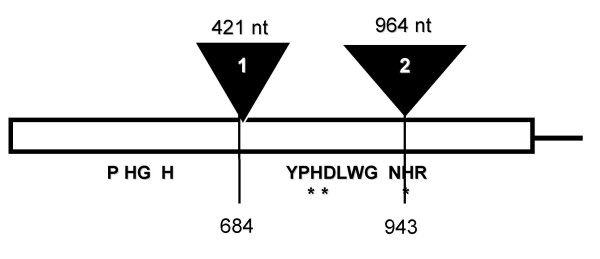
Genomic organization of *gF6H *(Adapted from [14])

In order to isolate the genomic region coding for F6H, genomic *C. americanum *DNA was used as a template for amplification reactions with primers designed to the outermost regions of the *cF6H *sequence. This produced a fragment of ~3.1 kbp (*gF6H*) that contained two potential intronic sequences (Figure 5). The first intron, 421 bp long is centrally located at position 684 of *cF6H*, whereas the second intron is significantly longer, 964 bp and is located towards the 3'-end of the gene at position 943.

**Figure 6:**
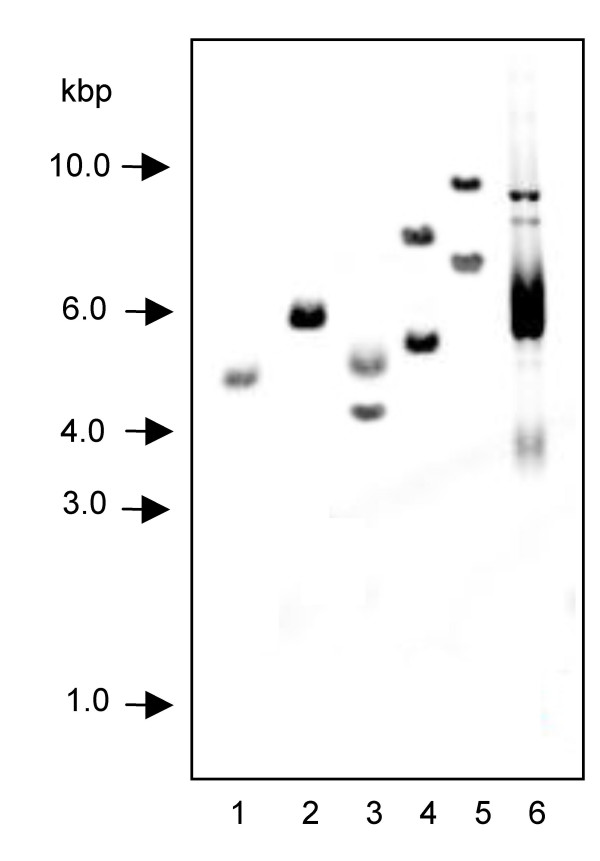
Southern blot of *C. americanum *genomic DNA Lanes: 1, *Bam*HI, 2, *Eco*RI, 3, *Kpn*I, 4, *Nco*I, 5, *Xho*I, 6, Undigested vector containing probe sequence

Southern analysis of genomic DNA, probed with the *cF6H *partial ORF, gave rise to single bands in *Bam*HI- and *Eco*RI-digested genomic DNA (Figure 6). The internal sequence of *gF6H *does not contain any recognition sites for the above enzymes; however, one recognition site is located within the known sequence for *Kpn*I, *Nco*I, *Xho*I. Two major bands were detected in these lanes at estimated sizes ranging from ~4.3 to 10.0 kbp. These results indicate that *F6H *is present as a single copy gene in *C*. *americanum*, particularly since potential tandem *F6H *repeats would have resulted in the observation of fragments larger than 6.2 kbp, which were not present in lane 1. Under conditions of reduced stringency, certain lanes exhibited more than the two expected bands (data not shown), indicating the existence of related sequences within the genome.

The ORFs of the clones isolated using different approaches were identical in sequence, indicating that they represent the same protein. This is in agreement with the results obtained from Southern analysis suggesting that *F6H *is present as a single copy in the *C. americanum *genome. The deduced amino acid sequence of *cF6H *exhibits homology to other ODDs, particularly at the C-terminal, and this conservation can be extended to the organization of the gene as a whole. The fact that *gF6H *possesses two introns at conserved locations for ODDs [14], suggests that ODD sequences arose through divergence from a common ancestor. The single copy nature of this enzyme also suggest a regulatory role in polymethylated flavonol biosynthesis and may be significant, in relation to a second hydroxylation that occurs at the 2'-position of the TMQ intermediate (Figure 1). A comparable reaction was recently reported to be catalyzed by a cytochrome P450-dependent monooxygenase involving the 2'-hydroxylation of isoflavones in *Medicago truncatula *[15]. It has been proposed that the cytosolic enzymes involved in the sequential methylation of Chrysosplenium flavonoids are organized on the surface of a multienzyme aggregate, thus allowing for a more efficient regulation of the pathway as a whole [16]. It is likely that the F6H is a component of this enzyme complement, as it introduces a hydroxyl group at position 6 of 3,7,4'-trimethylquercetin for subsequent *O*-methylation at this position by a flavonol 6-*O*-methyltransferase (F6OMT). Immunolocalization of both enzymes, F6H and F6OMT, should provide further evidence in support of this view.

**Figure 7:**
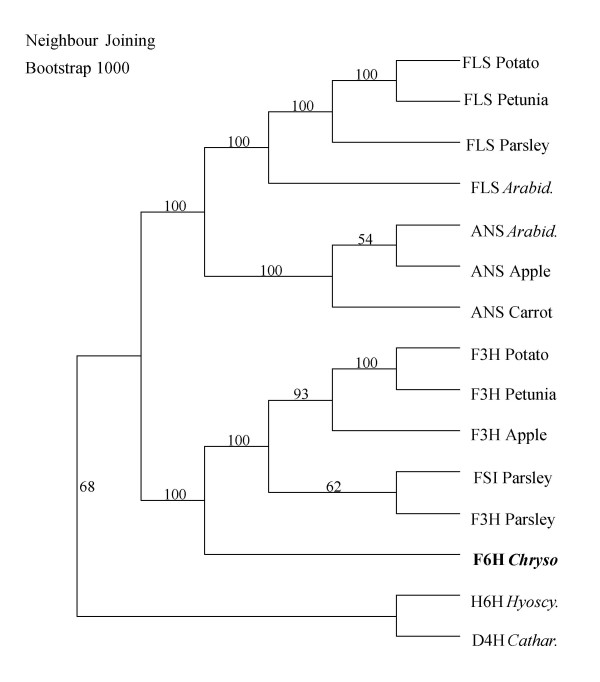
Phylogenetic analysis of *cF6H *Accession numbers for above genes: Potato FLS (X92178); Petunia FLS (Z22543); Parsley FLS (AY230249); *Arabidopsis *FLS (U84259); *Arabidopsis *ANS (U70478); Apple ANS (X71360); Carrot ANS (AF184273); Potato F3H (AY102035); Petunia F3H (X60512); Apple F3H (X69664); Parsley Flavone synthase (AY230247); Parsley F3H (AY230248); *Hyoscyamus *hyoscyamine 6β-hydroxylase (M62719); *Catharanthus *desacteoxyvindoline 4-hydroxylase (U71604).

*cF6H *from *C. americanum *exhibits highest similarity, at the amino acid level, to various *F3H *homologs. Phylogenetically, it is evident that homologous genes encoding biochemically related proteins in single pathways are clustered into related subgroups (Figure 7), perhaps as a result of gene duplication and divergence. Genes encoding proteins in the same pathway of a given species, although not within the same subgroup, exhibit expectedly higher identity than unrelated genes. *cF6H *clusters with the *F3H *group of genes, although at a position distal to those homologs from other species, thus suggesting an evolutionary relationship with this particular subgroup of biosynthetically related enzymes. It is evident from Figure 7 that flavonol ODDs cluster into 2 distinct clades; the first consisting of enzymes with a narrow substrate specificity including F3H, FSI and F6H, whereas the second is comprised of FLS and ANS, both possessing broad substrate specificity as has been previously described [12,17,18]. It seems that based on biochemical and phylogenetic considerations, *Chrysosplenium *F6H exhibits high substrate and position specificity.

At the phylogenetic level, ODDs comprise a superfamily with numerous subgroups whose subsets are defined by shared motifs in the encoded proteins, such as those involved in flavonoid biosynthesis. These motifs often comprise the active site of the enzyme and/or the binding domains for substrates and cofactors. In addition, the degree of similarity between genes encoding dioxygenases with different substrate preferences suggests a common reaction mechanism. The molecular characterization of F6H and related ODDs could identify potential commonalities in structure or reaction mechanisms, as well as reveal motifs or residues that may determine reaction type and/or substrate preference. When aligned with related sequences, *cF6H *exhibits a high degree of conservation of the motifs required for iron and cosubstrate binding, whereas other regions show little or no identity and are presumed, most likely, to contribute to differences in substrate specificity and/or reaction type. This applies particularly to the variable N-terminal and a region where a gap insertion within an ODD sequence motif is required for proper alignment with other flavonoid biosynthetic enzymes. Further evidence of such distinctness is the fact that, although F6H clusters with the F3H group of flavonoid dioxygenases (Figure 7), it appears to have evolved paraphyletically from a common ancestor with respect to this group of genes.

## Conclusions

A novel ODD cDNA involved in the 6-hydroxylation of partially methylated flavonols was isolated and characterized from *C. americanum*. The substrate specificity observed with the recombinant F6H compares well with the native enzyme [3]; displaying a specificity for position 6 of partially methylated flavonols, with 3,7,4'-trimethylquercetin being the preferred substrate. The fact that it hydroxylates position 6 of the aromatic ring A of a partially methylated flavonol contrasts with other aromatic hydroxylations which are commonly catalyzed by cytochrome P450 monooxygenases, such as the flavonol 8-hydroxylase of *Tagetes patula *[19] and the flavonoid 6-hydroxylase of soybean involved in isoflavone biosynthesis [20]. Given that both the native and recombinant F6H exhibit a preference for relatively hydrophobic substrates further distinguishes this protein from other dioxygenases characterized to date, that are known to accept relatively polar flavonoid aglycones [21-24]. Further evidence of such distinctness is the fact that, although *cF6H *clusters with the *F3H *group of flavonoid dioxygenases (Figure 7), it appears to have evolved in a paraphyletic fashion with respect to this group of genes. General flavonoid biosynthesis may have arisen from a multifunctional dioxygenase, since F3H and F6H seem to share a common ancestor, but significant divergence has since occurred resulting in the evolution of this novel activity.

A clone with full enzyme activity, which can be isolated based on the *cF6H *sequence provided fresh tissue is available, can be used to assess the versatility of this enzyme with respect to substrate preference through site-directed mutagenesis, as well as to assess its potential applications in metabolic engineering.

## Methods

### Materials

*Chrysosplenium americanum *(Schwein x Hooker; Saxifragaceae), was collected from St. Anicet, Québec, and was maintained in the greenhouse under conditions simulating its natural habitat regarding light intensity, temperature and humidity.

Flavonol substrates were from our laboratory collection. All other chemicals were of analytical reagent grade. Buffers: Assay buffer, 50 mM Tris-HCl (pH 7.3), 10 mM DTT, 150 mM NaCl, 10% glycerol (v/v); Wash solution I, 2 × SSC, 0.1% SDS (w/v); Wash solution II, 0.1 × SSC, 0.1% SDS (w/v); Blocking solution, 5% SDS (w/v), 125 mM NaCl, 25 mM sodium phosphate (pH 7.2); Blot washing solution, 0.5% SDS (w/v), 12.5 mM NaCl, 2.5 mM sodium phosphate (pH 7.2). FPLC buffers were filtered, degassed and stored at 4°C.

### Isolation of an F6H clone

The ligand-affinity purified F6H protein [3] was subjected to digestion and sequencing at the Harvard Microchemistry Facility using microcapillary, reverse-phase HPLC nano-electrospray tandem mass spectrometry (MS/MS) on a Finnigan LCQ quadrupole ion trap mass spectrometer. The MS/MS spectra obtained were correlated with known sequences using the Sequest algorithm developed at the University of Washington [25,26].

A *C. americanum *cDNA Lambda UniZap XR library was screened by PCR as described in [27] with 5 μL of the cDNA library (representing 1 × 10^6 ^pfu) per reaction. The following degenerate oligonucleotide primers were derived from two internal peptide sequences obtained from the native protein, Micro1 and Micro2;

Micro1For-5'-GCTGGATCCTCCTTCATATA-3';

Micro1Rev-5'-(GC)GATAC(AT)GG(TC)AA(TC)(CG)TTAG(ATGG(CT)TATAC-3';

Micro2For-5'-AATTGTTGAAGCATGTGAAG-3';

Micro2Rev-5'-(TC)GGGGTTAG(AG)AG(TC)GT(AC)CG(AG)AA-3';

T3 + 6 - 5'-GCT CGA AAT TAA CCC TCA CTA AAG GG-3'

T7 + 6 - 5'-GAA TGG TAA TAC GAC TCA CTA TAG GGC G-3'

Primer combinations: i) Micro1Rev and Micro2For; ii) Micro1For and T3; iii) Micro1Rev and T7; iv) Micro2For and T3; v) Micro2Rev and T7. PCR products were subjected to DNA sequencing for characterization after cloning into the pGEM-T vector (Promega). DNA sequencing was carried out using the dideoxy-mediated chain termination method [28]. Fragment F6H·1 was amplified using a mixed primer set, T7 and Micro1Rev, and contained the peptide sequence to which the primer was designed, as well as certain conserved ODD motifs and a second peptide sequence. In order to isolate full-length putative F6H clones, new sets of primers were designed specifically to the ends of the F6H·1 sequence and the screening procedure was repeated with vector primers under stringent conditions. Alternatively, the bacteriophage cDNA library was screened as described in [29], using the F6H·1 fragment (602 bp) as an oligonucleotide probe. The near-full-length cDNA fragment isolated that putatively encoded the F6H was termed *cF6H*. Given the lack of fresh *C. americanum *tissue and its disappearance from its natural habitat, for use in 5'-rapid amplification of cDNA ends (RACE) experiments or primer extension, genomic DNA-based techniques such as inverse PCR and GenomeWalker (ClonTech) were employed in attempts to isolate the remaining 5'-end of the putative F6H sequence. Nevertheless, neither of these approached proved successful.

To isolate the genomic region coding for the *cF6H*, *C. americanum *genomic DNA (0.1 to 1.0 μg) was employed as a template for PCR using primers designed to the ends of the *cF6H *sequence. Putatively positive fragments were subsequently cloned into the pGEM-T vector for DNA sequencing.

To determine the copy number of *F6H*, genomic DNA (15 μg) was digested overnight and subjected to Southern analysis according to [30]. The probe, *cF6H*, was labeled using the BioPrime Kit (Amersham) designed for the preparation of biotinylated nucleotide probes for blotting with the IRDye800-conjugated Streptavidin (Rockland Immunochemicals) as the detection system. The membrane was pre-hybridized using UltraHyb-OS (Ambion) at 42°C for 2–3 h. Hybridization was carried out with denatured, biotinylated probe solubilized in a fresh aliquot of UltraHyb-OS overnight at 42°C. The membrane was washed according to manufacturer's instructions using wash solutions I and II. The membrane was blocked with blocking solution for one hour at room temperature, prior to probing with IRDye800-labelled streptavidin (1:10,000) in blocking solution for 1 h at room temperature. The membrane was washed with blot washing solution three times, 15 min each, prior to detection.

### Cloning and expression strategies

The cDNA fragment encoding the near-full length F6H, *cF6H *was cloned into an *E. coli *expression system (pTrc-His; Invitrogen) The fragment was amplified by PCR using gene-specific primers, containing *Bam*HI and *Hin*dIII recognition sites, respectively. The DNA insert is positioned downstream and in-frame with a sequence encoding the (His)_6_-tag and an enterokinase cleavage recognition site. The pTrc-His-F6H construct was transformed into Top 10 cells by heat shock, and selected on ampicillin (50 μg/mL) containing media. Recombinant protein production in *E. coli *strain Top 10 was induced by the addition of 1 mM isopropyl-β-D-thiogalactopyranoside (IPTG) for 4 h at 37°C. After sonication of the cell pellet and centrifugation, the supernatant was assayed for F6H enzyme activity or purified on Ni-NTA resin (Qiagen). The affinity-purified protein was eluted in presence of 250 mM imidazole and subjected to buffer exchange on a PD-10 column against the assay buffer. Enzyme assays and analysis of reaction products were carried out as previously described [3].

Heterologous expression in the *Pichia pastoris *expression system (EasySelect pPicZ-His; Invitrogen) can be either intracellular (pPicZ) or secreted (pPicZα), depending on the presence of an in-frame signal peptide. The *cF6H *fragment was amplified by PCR using gene-specific primers, containing *Kpn*I and *Sac*II recognition sites, respectively. For the pPicZ construct, the initiation ATG was part of the yeast consensus sequence (AATAATGTCT) included in the 5' gene-specific cloning primer. For the pPicZα construct, the insert was cloned in-frame with the N-terminal signal sequence and C-terminal His-tag. The pPicZ-cF6H and pPicZα-cF6H constructs were transformed into *Pichia *cells by electroporation and putative multi-copy recombinants were selected on Zeocin-containing media according to manufacturer's instructions (Invitrogen).

Recombinant F6H production was induced by the addition of 0.5% methanol (in the absence of glucose) every 24 h for 3 to 4 days. A buffered culture medium (BMGY/BMMY; containing 100 mM potassium phosphate, pH 8.0) was used for cell growth and protein induction in order to enhance protein stability and limit enzyme inactivation. In the case of intracellular recombinant protein production, cells were lysed using glass beads and extracts were prepared according to manufacturer's instructions (Invitrogen). Secreted proteins were collected and concentrated by ammonium sulfate precipitation (35 to 70% saturation), and used for activity assays after desalting or for subsequent affinity purification.

Recombinant proteins obtained from both prokaryotic and eukaryotic expression systems were subjected to SDS-PAGE [31] and Western blot [32] analysis using chemiluminescent (HRP; Amersham) or IRDye800 (Li-Cor) detection.

### Phylogenetic analyses

Fifteen ODD genes involved in secondary metabolism where biochemical information was available, belonging to different plant species, were selected from the PubMed and GenBank searches. The amino acid sequences were aligned using CLUSTAL-W [33] and PHYLIP output format. The data was transferred into MacClade 4.03 [34] for visual inspection and manual editing prior to analysis by a phylogenetic tree building and analysis program, PAUP 4.0, beta 4 [35]. The optimality criterion employed for the distance method was Neighbor-Joining [36]. The aligned amino acid sequences were analyzed using a heuristic search algorithm with 1000 random addition sequences. Bootstrapping analysis was carried with heuristic search based on 1000 replicates and the distance measure was set to be equal to mean character difference.

## List of abbreviations

*cF6H *F6H cDNA

F6H flavonol 6-hydroxylase

*gF6H *genomic F6H

ODD 2-oxoglutarate-dependent dioxygenase

α-KG α-ketoglutarate

ORF open reading frame

rF6H recombinant F6H

rF6Hb recombinant F6H expressed in bacteria (*E. coli*)

rF6Hy recombinant F6H expressed in yeast (*P. pastoris*) – intracellular

rF6Hys recombinant F6H expressed in yeast (*P. pastoris*) – secreted

TMQ 3,7,4'-trimethylquercetin

TMQg 3,7,4'-trimethylquercetagetin

## Authors' contributions

DA carried out experiments in the project, participated in the design and coordination of the project, as well as the writing of the manuscript. RKI conceived of the project and contributed to the writing of the manuscript. All authors read and approved the final manuscript.

## Figures and Tables

**Table 1:** Specific activity of recombinant F6H proteins^*a*^

Step	Specific Activity (pkat/mg)
rF6Hb	
Cell Lysate	0
Ni-NTA	0.091
Ni-NTA (30°C)	0.087
Ni-NTA + Superose 12	0.103
Superose 12	0.075
Cleavage of (His)_6_-tag after Ni-NTA	0.044
rF6Hy (intracellular)	
Cell Lysate	0
Ni-NTA	0.17
Ni-NTA + Superdex 75	0.21
Superdex 75	0.087
rF6Hys (secreted)	
(NH_4_)_2_SO_4 _(35–70%)	0
(NH_4_)_2_SO_4 _+ Ni-NTA	0.11
(NH_4_)_2_SO_4 _+ Ni-NTA + Superdex 75	0.08
Superdex 75	0.04

^*a *^All assays were carried in the presence of assay buffer, 5 μM 3,7,4'-trimethylquercetin and ODD cofactors using the F6H enzyme assay as described in [3].

**Table 2:** Substrate specificity of the recombinant F6H Both rF6Hb and rF6Hy were purified on Ni-NTA resin then Superose 12, and fractions were assayed with the indicated substrates, using the direct enzyme assay.

Substrate^*a*^	Relative enzyme activity (%)	
	rF6Hb ^*b*^	rF6Hy ^*c*^
3,7,4'-Trimethylquercetin	100	100
3,7-Dimethylquercetin	56	63
3,7,3',4'-Tetramethylquercetin	11	21
3-Methylquercetin	0	4

^*a *^No F6H activity was detected with kaempferol, quercetin, myricetin, naringenin, eriodyctyol, apigenin or luteolin when used as substrate, regardless of concentration. Rhamnetin (7-*O*-methylquercetin, 17% at 50 μM), tamarixetin (4'-*O*-methylquercetin, 13 % at 50 μM) and isorhamnetin (3'-*O*-methylquercetin, 4 % at 50 μM)

^*b *^Estimated as 0.10 pkat/mg for 100% activity and 5 μM substrate.

^*c *^Estimated as 0.19 pkat/mg for 100% activity and 5 μM substrate.
